# Discovery of the Potentiator of the Pore-Forming Ability of Lantibiotic Nisin: Perspectives for Anticancer Therapy

**DOI:** 10.3390/membranes12111166

**Published:** 2022-11-20

**Authors:** Dayana N. Chernyshova, Alexander A. Tyulin, Olga S. Ostroumova, Svetlana S. Efimova

**Affiliations:** Laboratory of Membrane and Ion Channel Modeling, Department of Molecular Physiology of the Cell, Institute of Cytology of Russian Academy of Sciences, Tikhoretsky 4, 194064 Saint Petersburg, Russia

**Keywords:** lantibiotic, nisin, ion channel, lipid bilayers, cardiolipin, membrane dipole potential, phloretin

## Abstract

This study was focused on the action of lantibiotic nisin on the phospholipid membranes. Nisin did not produce ion-permeable pores in the membranes composed of DOPC or DOPE. The introduction of DOPS into bilayer lipid composition led to a decrease in the threshold detergent concentration of nisin. An addition of nisin to DOPG- and TOCL-enriched bilayers caused the formation of well-defined ion pores of various conductances. The transmembrane macroscopic current increased with the second power of the lantibiotic aqueous concentration, suggesting that the dimer of nisin was at least involved in the formation of conductive subunit. The pore-forming ability of lantibiotic decreased in the series: DOPC/TOCL ≈ DOPE/TOCL >> DOPC/DOPG ≥ DOPE/DOPG. The preferential interaction of nisin to cardiolipin-enriched bilayers might explain its antitumor activity by pore-formation in mitochondrial membranes. Small natural molecules, phloretin and capsaicin, were found to potentiate the membrane activity of nisin in the TOCL-containing membranes. The effect was referred to as changes in the membrane boundary potential at the adsorption of small molecules. We concluded that the compounds diminishing the membrane boundary potential should be considered as the potentiator of the nisin pore-forming ability that can be used to develop innovative formulations for anticancer therapy.

## 1. Introduction

One of the main problems of successful anticancer therapy is multidrug resistance, which seriously limits the effectiveness of the chemotherapeutic agents used and plays a significant role in tumor metastasis [1,2,3]. The emergence of resistant cells requires the development of new strategies to combat various types of cancer and the search for new chemotherapeutic compounds. Given that there are fundamental differences between the membranes of normal and tumor cells [4,5,6,7], targeting lipids, the content of which changes significantly during malignant transformation of cells, is a promising approach for the development of anticancer drugs. In this regard, antimicrobial peptides attract the most attention. They are able to induce selective apoptotic death and reduce cell proliferation in various cancer lines [8]. The role of changes in the properties of the plasma and intracellular membranes of transformed cells, especially permeabilization resulting from pore formation, in the antitumor effect of antimicrobial peptides remains poorly understood.

Nisin is a cationic, thermo stable and hydrophobic lantibiotic composed of 34 amino acids; it contains four nonstandard amino acid residues, didehydroalanine and didehydrobutyrin, and five rings formed by one lanthionine and four β-methyllanthionine bridges [9]. It is known that nisin inhibits the growth of invasive mammary ductal adenocarcinoma (MCF-7) and human hepatocellular carcinoma (HepG2) cell lines with an IC_50_ of about 100 μM [10,11]. Maher and McClean found the significant cytotoxicity of nisin A against colorectal adenocarcinoma cell lines, HT29 and Caco-2, with IC_50_ of about 90 and 115 μM, respectively [12]. Nisin also demonstrates a cytotoxic activity in vitro and in vivo against various lines of human oral squamous cell carcinoma [8,13]. At the concentrations from 6 to 25 μM, nisin causes a significant increase in apoptosis due to an increase in calcium; moreover, it is characterized by the high selectivity towards transformed cells compared to the primary keratinocytes from the oral cavity [8].

The mechanisms of the antitumor activity of nisin are debatable. Some authors associate the cytotoxic effect of nisin with a violation of the integrity of cancer cell membranes [14]. It has been shown that nisin increases the fluidity of the lipid membranes and reduces the dipole potential of lipid bilayers mimicking neuroblastoma cell membranes [15]. The authors associated the disordering effect of nisin with its ability to destroy lipid rafts, the number of which is increased in the membranes of transformed cells compared to non-malignant cells [16].

Cardiolipin is involved in different stages of the mitochondrial apoptotic process [17]. Thus, cardiolipin should be considered as one of the apoptosis triggers, and it makes the study of nisin activity in cardiolipin-enriched membranes to be of key importance for understanding the mechanisms of lantibiotic anticancer action.

The molecular mechanisms of antibacterial action of nisin include the lantibiotic interaction with peptidoglycan precursor lipid II biosynthesis [18], pore formation in the bacterial membranes, and, consequently, lysis of the pathogenic cells [19,20,21]. Using the circular dichroism it was demonstrated that nisin:lipid II stoichiometry in the pore complex is about 8:4 [22]. Using model lipid membranes, Wiedman et al. demonstrated that nisin is able to form pores in the diphytanoylphosphatidylcholine membranes with and without lipid II [23]. Moreover, the authors concluded that the presence of lipid II in the diphytanoylphosphatidylcholine membrane stabilizes the pores formed by nisin. Other authors found that nisin has a greater affinity to negatively charged bilayers than to zwitterionic ones [24,25], that is consistent to the studies showing that nisin predominantly forms pores in negatively charged bilayers composed of dioleylphosphatidylcholine and cardiolipin or dioleylphosphatidylcholine and phosphatidylserine [26,27]. The authors explained that the positively charged nisin electrostatically interacts with negatively charged head groups of lipid molecules. Lins et al. showed that nisin curves the surface of the phosphatidylglycerol layer suggesting it could induce micelle formation [28].

The aim of this study was to investigate the molecular mechanisms of the actions of nisin on the phospholipid membranes and to find the potential potentiators of the lantibiotic pore-forming activity using small molecules of various chemical natures. The dependence of the pore-forming ability of nisin on the type of negatively charged lipid was demonstrated. The threshold concentrations of nisin caused the formation of the single channels, the microscopic transmembrane current, and the disintegration of the lipid bilayers were determined. The analysis of the dependence of the steady-state nisin-induced current flowing thought cardiolipin-enriched membranes on the concentration of the lantibiotic allowed to determine the number of the nisin molecules participating in the pore formation: it was close to 2. Phloretin, capsaicin, RH 421, and tetracaine, were used to modulate the pore-forming ability of nisin. Phloretin and capsaicin were found to potentiate the activity of the lantibiotic.

## 2. Materials and Methods

### 2.1. Chemical Reagents

KCl, KOH, HEPES, EDTA, DMSO, nonactin, pentane, ethanol, di-8-ANEPPS, calcein, sephadex G-50, triton X-100, nisin from *Lactococcus lactis*, phloretin (2′,4′,6′-trihydroxy-3-(4-hydroxyphenyl) propiophenone), capsaicin (8-methyl-N-vanillyl-*trans*-6-nonenamide), tetracaine (4-(butylamino)benzoic acid 2-(dimethylamino)ethyl ester), and RH 421 (N-(4-sulfobutyl)-4-(4-(4-(dipentylamino)phenyl)butadienyl) pyridinium) were purchased from Sigma-Aldrich Company Ltd. (Gillingham, UK). A total of 0.1 M KCl solutions were buffered using 10 mM HEPES-KOH at pH 7.4. Synthetic 1,2-dioleoyl-*sn*-glycero-3-phosphocholine (DOPC), 1,2-dioleyl-*sn*-glycero-3-phosphoethanolamine (DOPE), 1,2-dioleyl-*sn*-glycero-3-phosphoserine (DOPS), 1,2-dioleyl-*sn*-glycero-3-phospho-(1′-*rac*-glycerol) (DOPG), and 1,1′,2,2′-tetraoleoyl cardiolipin [4-(dipyrrometheneboron difluoride)butanoyl] (TOCL) were obtained from Avanti Polar Lipids^®^ (Avanti Polar Lipids, Alabaster, AL, USA).

Dulbecco’s Modified Eagle Medium (DMEM), gentamycin solution 10 mg/mL for cell culture, Versene solution 0.02 % Trypsin solution 0.25 %. were purchased from Biolot (Saint-Petersburg, Russia), Fetal Bovine Serum (FBS) was purchased from DIAEM (Moscow, Russia). To prepare the nisin solution, it was dissolved in 0.02 N HCl to get a concentration of 5 mM. The phloretin solution was dissolved in DMSO up to a concentration of 100 mM. A human hepatocellular carcinoma (HepG2) cell line was provided by the shared research facility «Vertebrate cell culture collection» (Agreement #075-15-2021-683). Plates and dishes for cell culture were purchased from Jet Biofil (Guangzhou, China).

All experiments were performed at room temperature (25 °C).

### 2.2. Reconstitution of Nisin into Planar Lipid Bilayers

Virtually solvent-free planar lipid bilayers were prepared using a monolayer-opposition technique [29] on a 50-µm diameter aperture in a 10-µm thick Teflon film separating the two (*cis*- and *trans*-) compartments of the Teflon chamber. Lipid bilayers were made from pure DOPC and DOPE or mixtures of DOPC/DOPS (50/50 mol%), DOPC/DOPG (50/50 mol%), DOPC/TOCL (50/50 mol%), DOPE/DOPS (50/50 mol%), DOPE/DOPG (50/50 mol%), and DOPE/TOCL (50/50 mol%) and bathed in 0.1 M KCl 10 mM HEPES at pH 7.4. After the membrane was completely formed and stabilized, nisin from stock solution in water (pH 3.0) was added to the *cis*-side of the chamber up to obtain a final concentration ranging from 0.5 μM to 2.2 mM.

The dependence of the steady-state nisin-induced transmembrane current on the concentration of lantibiotic was characterized by the threshold concentration of lantibiotic required for the appearance of pores (*C_tr_*), the threshold concentration of lantibiotic required to reach of the maximum steady-state macroscopic current (*C*|_*Imax*_) and the maximum steady-state macroscopic current (*I_max_*). A criterion for reaching a steady-state current level was d*I*/d*t* ≤ 0.

Small molecules (phloretin, capsaicin, tetracaine, and RH 421) were added to both sides of the membranes up to 20, 400, 500, and 10 μM, respectively. A steady-state transmembrane current induced by nisin (*I_agent_*/*I*^0^*_control_*) was used to assess the alteration in the pore-forming activity of nisin by two-sided addition of small molecules.

Ag/AgCl electrodes with 1.5% agarose/2 M KCl bridges were used to apply *V* and measure the transmembrane current (*I*). “Positive voltage” refers to the case in which the *cis*-side compartment is positive with respect to the *trans*-side. Transmembrane current was measured using an Axopatch 200B amplifier (Molecular Devices, LLC, Orleans Drive, Sunnyvale, CA, USA) in the voltage clamp mode. Data were digitized using a Digidata 1440A and analyzed using pClamp 10.0 (Molecular Devices, LLC, Orleans Drive, Sunnyvale, CA, USA) and Origin 8.0 (OriginLab Corporation, Northampton, MA, USA). Data were acquired at a sampling frequency of 5 kHz using low-pass filtering at 1 kHz, and the current tracks were processed through an 8-pole Bessel 100-kHz filter.

### 2.3. Measurements of the Changes in the Membrane Boundary and Its Component Dipole Potential

The membrane boundary potential (φ*_b_*) consists of the surface potential (φ*_s_*), related to the presence of the charged lipids or molecules on the membrane surface, and the dipole potential (φ*_d_*), related to the dipolar components of lipids, interface water and other adsorbed molecules [30,31,32].

The steady-state membrane conductance induced by K^+^-nonactin was modulated via a two-sided addition of small molecules (phloretin, capsaicin, tetracaine, and RH 421) from mM stock solutions in water or ethanol to the membrane-bathing solution (0.1 M KCl, 10 mM HEPES, pH 7.4) up to concentration of 20, 400, 500, and 10 μM, respectively. Lipid bilayers were made from mixture of DOPC/TOCL (50/50 mol%). The conductance of the lipid bilayers was determined by measuring *I* at a constant transmembrane voltage (*V* = 50 mV). The subsequent calculations were performed assuming that the membrane conductance (*G* = *I*/*V)* is related to the membrane boundary potential (φ*_b_*) by the Boltzmann distribution [33]:

(1) $$\frac{G_{m}}{G_{m}^{0}}=\exp(-\frac{q_{e}\Delta \varphi_{b}}{kT})$$

where *G_m_* and $G_{m}^{0}$ are the steady-state membrane conductance induced by K^+^-nonactin in the presence and absence of small molecules, respectively, *q_e_* is the electronic charge, *k* is the Boltzmann constant, and *T* is the temperature in Kelvins.

The dipole component of the bilayer boundary potential, φ*_d_*, was measured using a dipole-potential-sensitive lipid fluorescence probe di-8-ANEPPS [34,35]. Large unilamellar vesicles composed of DOPC/TOCL (50/50 mol%), containing 1 mol% di-8-ANEPPS, were prepared by extrusion using an Avanti Polar Lipids^®^ mini-extruder (Avanti Polar Lipids, Alabaster, AL, USA). Small molecules (phloretin, capsaicin, and tetracaine) were added to liposome suspension up to a concentration of 20, 400, and 500 μM, respectively, and had been incubated for 5 min before measurements. Steady-state fluorescence measurements were performed using Fluorat-02-Panorama spectrofluorometer (Lumex, Saint-Petersburg, Russia). The changes in the dipole potential of DOPC/TOCL (50/50 mol%) bilayers were estimated, as described in [35] using obtained values of *R*. The fluorescence excitation ratio *R* was defined as a ratio between the fluorescence intensities of di-8-ANEPPS at excitation wavelengths of 420 nm and 520 nm and at emission wavelength of 670 nm to avoid the influence of the elastic properties of the membrane [36,37].

### 2.4. Calcein Assay

Large unilamellar vesicles were prepared from pure DOPC, and mixtures of DOPC/DOPS (50/50 mol%), DOPC/DOPG (50/50 mol%), and DOPC/TOCL (50/50 mol%) by extrusion. Lipid stock in chloroform was dried under a gentle stream of nitrogen. Dry lipid film was hydrated by a buffer (35 mM calcein, 10 mM HEPES, pH 7.4). The suspension was subjected to five freeze–thaw cycles and then passed through a 100 nm Nuclepore polycarbonate membrane 13 times. The calcein that was not entrapped in the vesicles was removed by gel filtration with a sephadex G-50 column to replace the buffer outside the liposomes with a calcein-free solution (0.15 M KCl, 1 mM EDTA, 10 mM HEPES, pH 7.4). The calcein in vesicles fluoresces very poorly, because of strong self-quenching at millimolar concentration, while the fluorescence of disengaged calcein in the surrounding media correlates to the membrane permeability in the presence of nisin.

The degree of calcein released was determined using a Fluorat-02-Panorama spectrofluorimeter (Lumex, Saint-Petersburg, Russia). The excitation wavelength was 490 nm, and the emission wavelength was 520 nm.

The relative intensity of leaked calcein fluorescence (*RF*, %) was calculated, as described in [38,39]. Triton X-100 was added to a final concentration of 1% to each sample in order to completely disrupt liposomes, and the intensity after releasing the total amount of calcein from liposomes was measured.

Time-dependence of calcein fluorescence de-quenching induced by 100 μM nisin had been measured over 80 min.

### 2.5. Cell Culture

HepG2 cell line was maintained using DMEM with 10% FBS and 10 μg/mL Gentamycin. The media was stored at 4 °C between use. Cell lines were cultured in a 100 mm Petri dish and treated for adhesion culture at 37 °C in a humidified atmosphere with 5% CO_2_. Cell lines were reseeded when they reached 80–90% confluence.

### 2.6. Mitochondrial Potential

The cells for the assessment of mitochondrial potential activity were seeded in 24-well plates at a density of 20 × 10^3^ cells per well in 500 μL of the corresponded full supplemented media. After overnight incubation, the media were removed, and cells were treated with a maximum concentration of nisin, phloretin and its combination solutions in FBS-free media in accordance with the cell viability assay. Moreover, as additional controls irinotecan and FBS-free media with a pH, which mimics the pH in a well with nisin, were added (data not shown). Cells were incubated with compounds for 24 and 48 h at 37 °C in 5% CO_2_. Then, cells were stained with 400 nM MitoTracker^TM^ Red CMXRos (ThermoFisher Scientific, MA, USA) for 30 min. Once detached from the plate, the cells were washed in PBS 1x. A fluorescent flow cytometry assay was performed using CytoFLEX (Beckman Coulter, IN, USA). The experiments were performed in duplicates.

### 2.7. Statistical Analysis

The values of *C_tr_*, *C*|*_Imax_*, *I_max_*, Δφ*_b_* and *I_agent_*/*I*^0^_*control*_ were averaged from 3 to 10 independent experiments and presented as *mean* ± *s.d.* (*p* ≤ 0.05).

## 3. Results and Discussion

### 3.1. The Role of Negatively Charged Phospholipids in the Pore-Forming Ability of Nisin

**Figure 1:**
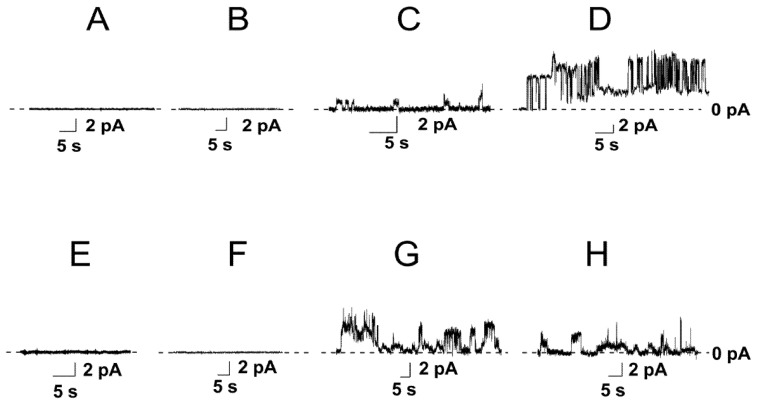
Examples of nisin-induced step-like current fluctuations through the membranes of different composition. The bilayers were composed of DOPC (**A**), DOPC/DOPS (50/50 mol%) (**B**), DOPC/DOPG (50/50 mol%) (**C**), DOPC/TOCL (50/50 mol%) (**D**), DOPE (**E**), DOPE/DOPS (50/50 mol%) (**F**), DOPE/DOPG (50/50 mol%) (**G**), and DOPE/TOCL (50/50 mol%) (**H**) and bathed in 0.1 M KCl, pH 7.4. The concentration of lantibiotics in bathing solution was equal to 2000 (**A**), 700 (**B**), 5 (**C**), 10 (**D**), 2000 (**E**), 600 (**F**), 60 (**G**), and 10 µM (**H**). *V* = 100 mV.

The specificity of nisin toward phospholipid membranes in the absence of its bacterial lipid adjuvant, lipid II, and the ability to form pores in the plasma and inner mammalian cell membranes might determine the lantibiotic cytotoxicity for cancer cells. Therefore, the influence of nisin on the ion permeability of planar bilayers of various phospholipid compositions was studied. It was shown that nisin was unable to form the transmembrane pores in the membranes composed of dioleoylphosphatidylcholine (DOPC) up to concentrations of 2000 µM (Figure 1A and Table 1). Further increase in the lantibiotic concentration led to membrane disintegration. As phosphatidylcholines are abundant for mammalian cell membranes [40] these data might indicate that nisin is unable to form the pores in plasma membranes of normal non-malignant cells.

According to the literature data the anionic phospholipids play an important role in nisin interaction to cell membranes [41,42]. Surprisingly, the introduction of negatively charged dioleoylphosphatidylserine (DOPS) into membrane lipid composition (DOPC/DOPS (50/50 mol%)) did not produce any ion-permeable pore-like events. However, the threshold concentration of detergent action of nisin was decreased to 700 µM (compared to 2000 μM for DOPC bilayers) (Table 1). Phosphatidylserine is asymmetrically distributed between the lipid monolayers of the plasma membrane and predominantly located in the inner membrane leaflet of the normal cells. However, in cancer cells, phosphatidylserine is often found at a high concentration in the outer monolayer of the plasma membrane [43,44,45,46]. In this regard, the detected selectivity of nisin toward phosphatidylserine (the decrease in the lantibiotic detergent threshold concentration) might indicate that the molecular mechanisms of the antitumor action of the lantibiotic might involve the nisin-induced increase in the permeability of the plasma membrane and penetration of the lantibiotic across the barrier to meet its intracellular targets. In line with this idea, Figure S1 (Supplementary Materials) demonstrates that nisin was able to induce an increase in the permeability of DOPC/DOPS (50/50 mol%) membrane for calcein, while it was ineffective toward the bilayers composed of pure DOPC.

**Figure 2:**
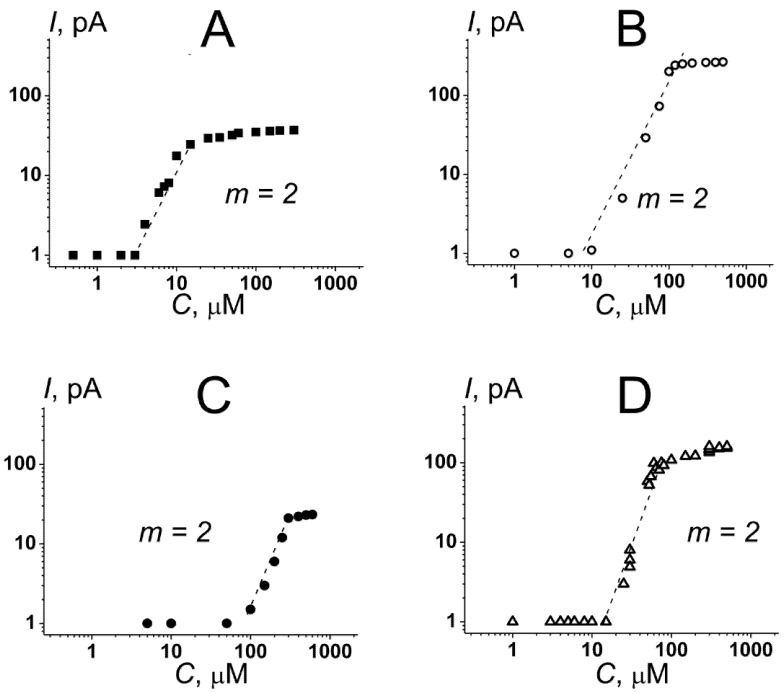
Dependence of the steady-state nisin-induced transmembrane current on the concentration of the lantibiotic in bilogarithmic coordinates. The membranes were made from (**A**) DOPC/DOPG (50/50 mol%), (**B**) DOPC/TOCL (50/50 mol%), (**C**) DOPE/DOPG (50/50 mol%), and (**D**) DOPE/TOCL (50/50 mol%) and bathed in 0.1 M KCl, pH 7.4. *V* = 100 mV. The slope (*m*) of the linear function fitting the growth region of the dependence characterizes the number of nisin molecules involved in the pore formation in membrane of appropriate composition.

The replacement of DOPS with dioleoylphosphatidylglycerol (DOPG) or cardiolipin (TOCL) led to appearance of the step-like current fluctuations at 5 and 10 µM, respectively (Table 1). Figure 1C demonstrated the typical record of transmembrane current fluctuations corresponding to the opening and closures of the nisin single ion pores in the DOPC/DOPG (50/50 mol%) membranes at a transmembrane voltage of 100 mV. The amplitude of nisin ion-permeable pores in DOPG-enriched bilayer varied in the range of 0.5–1.0 pA. The increase in the nisin concentration up to 25 µM caused by the enhancement of the number of the open pores in the bilyaer and produced the macroscopic transmembrane current (*I_max_*) of about 30 pA (Figure 2A and Table 1).

Subsequent increase in the lantibiotic concentration did not change the *I_max_*-value, and the disintegration of the DOPC/DOPG (50/50 mol%) membranes occurred at nisin concentrations of more than 750 µM (Table 1). Figure 2A demonstrates the dependence of the steady-state nisin-induced transmembrane current, flowing through the DOPC/DOPG (50/50 mol%) bilayer, on the aqueous concentration of lantibiotic. The slope of the linear regression of the growth region of *I*(*C*)-bilogariphmic plot was close to 2. This result suggested the oligomerization of nisin molecules during the formation of pores and an involvement of at least a dimer of lantibiotic molecules to the formation of a conductance unit. Figure 1D shows the step-like current fluctuations of different amplitudes induced by nisin in the DOPC/TOCL (50/50 mol%) membranes at a transmembrane voltage of 100 mV. The increase in the concentration of the lantibiotic from 10 to 100 µM led to a dose-dependent increase in the nisin-induced transmembrane current and reached the plateau (*I_max_*) of 250 pA at nisin concentrations more than 100 µM (Figure 2B and Table 1). Thus, the value of *I_max_* through the TOCL-containing membranes was 8-times higher than through the DOPG-enriched bilayers (Figure 2A vs. Figure 2B). The destruction of the DOPC/TOCL (50/50 mol%) bilayers was observed at lantibiotic concentrations of more than 600 μM (Table 1). Similar to the PG-enriched membranes the slope of the linear function fitting the growth region of the *I*(*C*)-bilogariphmic plot in the DOPC/TOCL (50/50 mol%) membranes was also close to 2 (Figure 2B). According to the literature data, cardiolipin is abundant for bacteria, and it is also a major lipid of the inner mitochondrial membrane [47,48,49]. The observed pore-formation by nisin in phosphatidylglycerol- and cardiolipin-containing bilayers might partially explain the lantibiotic antimicrobial effect by the perforation of the bacterial inner membrane. This assumption is in agreement with the findings of Verheul et al. [50] and Crandall and Montville [51] that found that the nisin-resistant strains of *Listeria monocytogenes* produce relatively less cardiolipin compared to nisin-sensitive strains. The involvement of cardiolipin in mitochondrial apoptosis pathway on the one hand, and the significant pore-forming ability of nisin in TOCL-enriched bilayers on the other hand, might also indicate the molecular mechanisms of the anticancer action of the lantibiotic. Moreover, according to [52,53], cardiolipin could be also missorted from mitochondria to the cell surface in the early stages of apoptosis, which acts as an in vivo trigger for the production of anticardiolipin antibodies. Therefore, another mechanism that is consistent with the action of nisin on plasma membrane as a primary target can be proposed.

To further investigate the action of nisin on the permeability of phospholipid membranes, we also examined the calcein leakage from vesicles composed of DOPC/DOPG (50/50 mol%) and DOPC/TOCL (50/50 mol%) (Supplementary Materials, Figure S1 and Table S1). Comparing DOPC and DOPC/DOPS (50/50 mol%) bilayers to DOPC/DOPG (50/50 mol%) and DOPC/TOCL (50/50 mol%) membranes we noticed that:

(1) The maximum leakage of the marker from vesicles composed of DOPC/DOPG (50/50 mol%) and DOPC/TOCL (50/50 mol%) was significantly higher than of DOPC and DOPC/DOPS (50/50 mol%) (Supplementary Materials, Figure S1 and Table S1). The results were consistent with the data of electrophysiological measurements (Figure 1).
(2) The time dependences in DOPC/DOPG (50/50 mol%) and DOPC/TOCL (50/50 mol%) bilayers were described by double-exponential functions instead of single-exponential functions used to fit the dependences in DOPC and DOPC/DOPS (50/50 mol%) membranes (Supplementary Materials, Figure S1 and Table S1). A biphasic nature of the curves was consistent with two different mechanisms of nisin action on phosphatidylglycerol- and cardiolipin-enriched membranes: in addition to the detergent action observed for all tested systems, nisin was able to form the pores exceptionally in DOPC/DOPG (50/50 mol%) and DOPC/TOCL (50/50 mol%) bilayers (Figure 1C,D).

Phosphatidylethanolamine is also found in bacterial membranes and its externalization from the inner leaflet of the plasma membrane is associated with the malignant transformation of cells [54,55,56]. Our data demonstrated that the replacement of DOPC with dioleylphosphatidylethanolamine (DOPE) in the bilayers formed from pure lipids or binary mixtures with DOPS was not accompanied by the appearance of ion-permeable pores (Figure 1E,F), while the increase in the lantibiotic concentration of more than 2000 and 600 μM caused the disruption of the DOPE and DOPE/DOPS (50/50 mol%) membranes, respectively (Table 1). Figure 1G shows the record of the step-like current fluctuations of different amplitudes induced by nisin in the DOPE/DOPG (50/50 mol%) membranes. Figure 2C and Table 1 demonstrate that the increase in the nisin concentration from 65 to 310 μM led to dose-dependent increase in the transmembrane current up to a steady-state level of about 30 pA, and the destruction of DOPE/DOPG bilayers occurred at lantibiotic concentrations of more than 650 µM. Nisin was also able to induce the ion pores of different amplitudes in DOPE/TOCL (50/50 mol%) bilayers at lantibiotic concentrations of more than 10 µM (Figure 1H). Figure 2D demonstrates that enlargement of nisin concentration from 15 to 80 µM caused a dose-dependent increase in the transmembrane current up to a steady-state macroscopic current of about 200 pA. The disintegration of the bilayer occurred at concentrations of more than 550 µM (Table 1). The slopes of the linear functions fitting the growth regions of *I*(*C*)-bilogariphmic plots for DOPC/DOPG (50/50 mol%) and DOPC/TOCL (50/50 mol%) bilayers were close to two (Figure 2C,D).

Analyzing the data presented in Table 1 and on Figure 2, one can conclude that the introduction of DOPE instead of DOPC in the TOCL-enriched membranes did not practically affect the pore formation and detergent activity of nisin. Wherein the nisin pore-forming ability was not the same in the bilayers composed of DOPC/DOPG and DOPE/DOPG mixtures: the replacement of DOPC with DOPE led to significant increases in *i_SC_*, *C_tr_* and *C*|*_Imax_*, while it did not influence the magnitudes of *m*, *I_max_* and *C_det_*. The increase in *C_tr_* and *C*|*_Imax_* in DOPE/DOPG membranes compared to DOPC/DOPG bilayers was in agreement with the observation of Crandall and Montville [49] that demonstrated that the nisin-resistant strain of *Listeria monocytogenes* ATCC 700,302 exhibited not only a decreased percentage of phosphatidylglycerol, but also an increased percentage of phosphatidylethanolamine.

The observed difference in the nisin action on DOPS- and DOPG-containing membranes (Supplementary Materials, Figure S1) contradicted the assumption that the negative charge of the membrane-forming lipids was the only factor regulating the lantibiotic interaction with phospholipid bilayers. The pore formation by nisin exclusively in DOPG- and TOCL-enriched membranes might indicate the key role of hydroxyl groups of glycerol residues. However, the observed difference between the sensitivity of bilayers composed of DOPC/DOPG (50/50 mol%) and DOPE/DOPG (50/50 mol%) mixtures to lantibiotic might be related to the alteration of the domain organization of the membranes, namely, the dependence of clustering of negatively charged lipids on the type of surrounding uncharged lipids. This possibility was consistent with the data by Lewis et al., which showed non-ideal mixing for a mixture of phosphatidylcholine and phosphatidylglycerol [57]. The poor miscibility was also demonstrated for phosphatidylethanolamine and cardiolipin mixture [58], although cardiolipin was not shown to form domains when mixed with phosphatidylcholine [59,60,61]. The domain organization of lipid bilayers might be further modified by the lantibiotic. Indeed, the model of clustering of anionic lipid in a bilayer composed of anionic and zwitterionic lipids in the presence of a cationic antimicrobial peptide and formation of defects in the surrounding area of the bilayer, increasing its permeability, was previously proposed [62].

### 3.2. Small Molecules Potentiating the Activity of Nisin in Cardiolipin-Containing Bilayers

Well-studied small molecules that can change the physicochemical properties of lipid bilayers were used to alter the pore-forming ability of nisin. Phloretin and capsaicin are known to decrease the membrane dipole potential of neutral phosphatidylcholine bilayers [63,64], while RH 421 and tetracaine are able to increase its value [63,65].

We measured the changes in the boundary potential of the DOPC/TOCL (50/50 mol%) membranes (φ*_b_*) and its dipole component (φ*_d_*) produced by the tested small molecules. Table 2 shows that the decrease in φ*_b_* due to the adsorption of 20 µM phloretin and 400 µM capsaicin was equal to approximately 90 and 100 mV, respectively. At the same time, 10 µM RH 421 and 500 µM tetracaine increased the φ*_b_*-value to about 110 and 50 mV, respectively (Table 2). Using the dipole-sensitive probe, di-8-ANNEPS, we also assessed the changes in dipole potential of DOPC/TOCL membranes induced by the introduction of the tested molecules. Table 2 shows that 20 µM phloretin and 400 µM capsaicin reduced the dipole potential of negatively charged membranes by about 100 and 80 mV, respectively, while 500 µM tetracaine did not practically change the φ*_d_*-value (the maximum increase in the bilayer dipole potential did not exceed 5 mV) (Table 2). Comparing the found values of Δφ*_b_* and Δφ*_d_*, we concluded that the changes in the DOPC/TOCL (50/50 mol%) membrane boundary potential produced by phloretin and capsaicin resulted from the alteration in its dipole component, while in the case of tetracaine φ*_b_,* it was probably affected by the surface component due to the adsorption of positively charged anesthetic molecules. The small percentage of RH 421 molecules in the ionized form (ρ) allowed the for changes in φ*_b_* to the alteration of φ*_d_* to be addressed similar to phloretin and capsaicin, although the spectrofluorimetric measurements using di-8-ANNEPS were not possible with the styryl dye.

**Figure 3:**
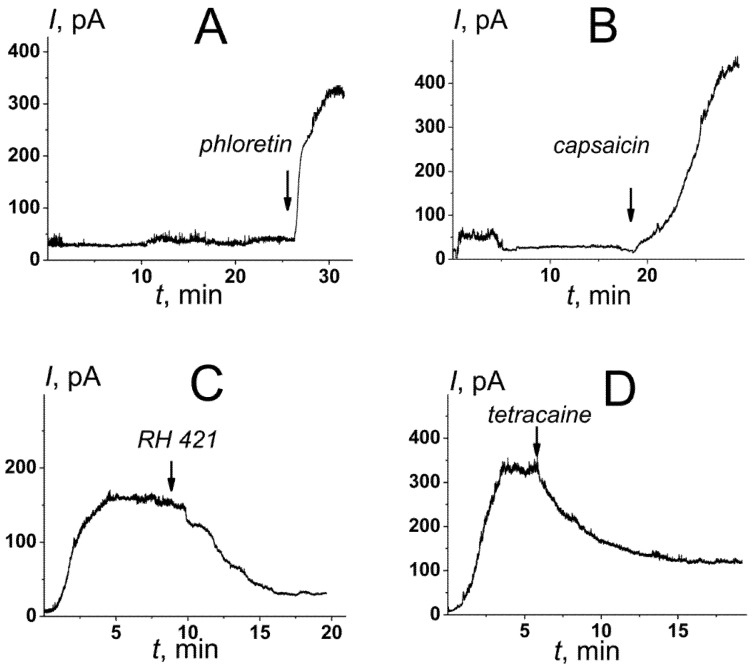
The effects of small molecules altering the membrane boundary potential on the steady-state bilayer conductance induced by *cis*-side addition of nisin. The moments of introduction of 20 μM phloretin (**A**), 400 μM capsaicin (**B**), 5 μM RH 421 (**C**), and 500 μM tetracaine (**D**) to the bilayer bathing solution are indicated by the arrows. The membranes were made from DOPC/TOCL (50/50 mol%) and bathed in 0.1 M KCl, pH 7.4. *V* = 100 mV.

Figure 3 demonstrates the effects of various small molecules, 20 μM phloretin, 400 μM capsaicin, 5 μM RH 421, and 500 μM tetracaine, on the macroscopic conductance induced by nisin in the DOPC/TOCL (50/50 mol%) membranes. Table 2 summarized the mean ratios between the nisin-induced steady-state transmembrane currents in the presence and absence of the tested compounds (*I_agen_*_t_/*I*^0^_*control*_). Phloretin and capsaicin led to a 5- and 11-fold increase in the steady-state nisin-induced transmembrane current, respectively (Figure 3A,B and Table 2), while the introduction of RH 421 and tetracaine caused 1.5–2-times decrease in the steady-state lantibiotic-induced transmembrane current (Figure 3C,D and Table 2). Significant correlation between *I_agent_*/*I*^0^_*control*_ and Δφ*_b_*-values was observed (the correlation coefficient by Pearson was equal to 0.74). Therefore, we concluded that the changes in the bilayer boundary potential due to the adsorption of the small molecules (due to the changes in its surface or dipole component) can modulate nisin pore-forming activity in the cardiolipin-enriched bilayers. Thus, the compounds diminishing the membrane boundary or dipole potential (in particular, phloretin and capsaicin) might be considered as potentiators of the anticancer action of nisin.

To examine the assumption that phloretin can be considered as a potential facilitator of anticancer action of nisin via depolarization of mitochondrial membrane, mitochondrial membrane potential was measured using flow cytometry (Supplementary Materials, Figure S2). Figure S2 demonstrates that a combination of nisin and phloretin significantly affected HepG2 cell line (right column), when both compounds separately did not possess such an effect (two middle columns) both at 24 (panel A) and at 48 h (panel B). Thus, the combination of nisin and phloretin had a huge impact on mitochondrial membrane potential, which indicates that phloretin might increase the apoptotic activity of nisin. The exact mechanism of facilitation should be explored further.

## 4. Conclusions

Summarizing the presented data, we concluded that:

(i) The observed decrease in the threshold concentration of nisin in the DOPS-containing bilayers compared to a membrane composed of DOPC might indicate that the antitumor action of the lantibiotic might involve the nisin-induced increase in the permeability of the plasma membrane of malignant cells at phosphatidylserine externalization.
(ii) The pore-formation by nisin in DOPG- and TOCL-enriched membranes might partially explain lantibiotic antimicrobial effects.
(iii) The pronounced pore-forming ability of nisin in TOCL-containing membranes might be a cause of its apoptotic action through mitochondrial pathway.
(iv) The ability of nisin to form pores depends on the distribution of the electrical potential on the membrane-solution interface: a decrease in the membrane boundary potential is accompanied by an increase in the pore-forming activity of the lantibiotic.
(v) Phloretin and capsaicin potentiate the pore-forming ability of nisin in TOCL-enriched membranes. The compound diminishing membrane dipole potential might be potential agonists of the antitumor action of nisin that creates the perspectives to develop innovative lantibiotic formulations.

## Figures and Tables

**Table 1:** The parameters characterizing the effects of nisin on the membrane of different phospholipid compositions.

Lipid Composition	Pore-Forming Ability				Detergent Activity
	*i_sc_*, pA	*C_tr_*, μM	*C|_Imax_*, μM	*I_max_*, pA	*C_det_*, μM
DOPC	–	–	–	–	2000 ± 50
DOPC/DOPS (50/50 mol%)	–	–	–	–	700 ± 10
DOPC/DOPG (50/50 mol%)	0.5 ÷ 1.0	5 ± 2	25 ± 3	30 ± 8	750 ± 20
DOPC/TOCL (50/50 mol%)	2 ÷ 29	10 ± 3	100 ± 5	250 ± 20	610 ± 10
DOPE	–	–	–	–	2050 ± 50
DOPE/DOPS (50/50 mol%)	–	–	–	–	600 ± 5
DOPE/DOPG (50/50 mol%)	1 ÷ 18	65 ± 5	310 ±15	30 ± 5	650 ± 10
DOPE/TOCL (50/50 mol%)	1 ÷ 27	12 ± 3	80 ± 15	195 ± 10	550 ± 15

*i_sc_*—the amplitude range of the nisin-induced single ion-permeable pores. *C_tr_*—the threshold concentration of the lantibiotic required to an appearance of ion-permeable pores. *C|_Imax_*—the threshold concentration of the lantibiotic required to reach of the maximum steady-state macroscopic current (*I_max_*). *C_det_*—the maximal threshold concentration required to disrupt the lipid bilayer.

**Table 2:** The effects of the small molecules on the electrical properties of DOPC/TOCL (50/50 mol%) membranes and nisin-induced transmembrane current.

*C*, μM	Small Molecules	*pKa ^#^*	*ρ*, % ^#^	Δφ*_b_*_,_ mV	Δφ*_d_*, mV	*I_agent_/I* ^0^ *_control_* ^a^
20	phloretin	7.96	−23	−86 ± 5	−99 ± 11	5.3 ± 1.3
400	capsaicin	4.25	0	−95 ± 11	−80 ± 12	11.3 ± 1.9
5	RH 421	5.96	−3	106 ± 11	–*	0.4 ± 0.1
500	tetracaine	8.42	+91	51 ± 6	4 ± 2	0.6 ± 0.1

Δφ*_b_* and Δφ*_d_*—the changes in the boundary and dipole potentials of the DOPC/TOCL (50/50 mol%) membranes, respectively. *I_modifier_*/*I_control_* ^a^—ratio between the transmembrane current induced by nisin in the presence and absence of the small molecules in the DOPC/TOCL (50/50 mol%) bilayers at *V* = 50 mV. ^#^—the values of *pKa* (the ionization constants) and percentage of molecules in the charged form at pH 7.4 were predicted by ChemAxon. *—was not measured due to crossing RH 421 and di-8-ANEPPS spectra.

## Supplementary Materials

The following supporting information can be downloaded at: https://www.mdpi.com/article/10.3390/membranes12111166/s1, Supplementary Figure S1. The dependence of relative fluorescence of calcein (*RF*) leaked from vesicles composed of DOPC (*black curve*), DOPC/DOPS (50/50 mol%) (*red curve*), DOPC/DOPG (50/50 mol%) (*green curve*) and DOPC/TOCL (50/50 mol%) (*blue curve*) on time (*t*). The lantibiotic was added into the liposomal suspension up to 0.1 mM at the zero time point. Supplementary Table S1. The parameters of exponential function fitting the time dependence of nisin-induced calcein leakage from liposomes of different phospholipid composition. Supplementary Figure S2. Histograms of the mitochondrial membrane potential of HepG2 cell line measured after 24 h (A) and 48 h (B) in the absence of any modifiers (control, *firth column*) and in the incubation with 1 mM nisin (*second column*), 0.1 mM phloretin (*third column*) and their combination 1 mM nisin and 0.1 mM phloretin (*fourth column*). The vertical axis represents a number of events, horizontal–fluorescent signal from the MitoTracker in APC (Allophycocyanin) channel. The red color signed “Mito” reflects the process of mitochondrial membrane depolarization, the black color is for cells with normal negative potential. The upper row of panel A and B represents cells excluding cell debris, lower row of panel A and B includes debris (“Mito(debris)”). Ten thousand events were recorded in total.
