# Supplementary material for: Discovery of the Potentiator of the Pore-Forming Ability of Lantibiotic Nisin: Perspectives for Anticancer Therapy
Source: Membranes (Basel). 2022 Nov 20;12(11):1166. doi: 10.3390/membranes12111166 (PMC9694817; doi:10.3390/membranes12111166)
Supplement: Supplementary file 1 [file membranes-12-01166-s001.zip › membranes-1986251-supplementary.pdf]

membranes

MDPI

Supplementary Material: Discovery of the Potentiator of the Pore-Forming Ability of Lantibiotic Nisin: Perspectives for Anticancer Therapy

Dayana N. Chernyshova, Alexander A. Tyulin, Olga S. Ostroumova and Svetlana S. Efimova *

Laboratory of Membrane and Ion Channel Modeling, Department of Molecular Physiology of the Cell, Institute of Cytology of Russian Academy of Sciences, Tikhoretsky 4, 194064 Saint Petersburg, Russia
* Correspondence: efimova@incras.ru

Citation: Chernyshova, D.N.; Tyulin, A.A.; Ostroumova, O.S.; Efimova, S.S. Discovery of the Potentiator of the Pore-Forming Ability of Lantibiotic Nisin: Perspectives for Anticancer Therapy. *Membranes* **2022**, *12*, 1166. https://doi.org/10.3390/membranes12111166

Academic Editor: Annarosa Arcangeli

Received: 7 October 2022
Accepted: 17 November 2022
Published: 20 November 2022

Publisher's Note: MDPI stays neutral with regard to jurisdictional claims in published maps and institutional affiliations.

Copyright: © 2022 by the authors. Licensee MDPI, Basel, Switzerland. This article is an open access article distributed under the terms and conditions of the Creative Commons Attribution (CC BY) license (https://creativecommons.org/licenses/by/4.0/).

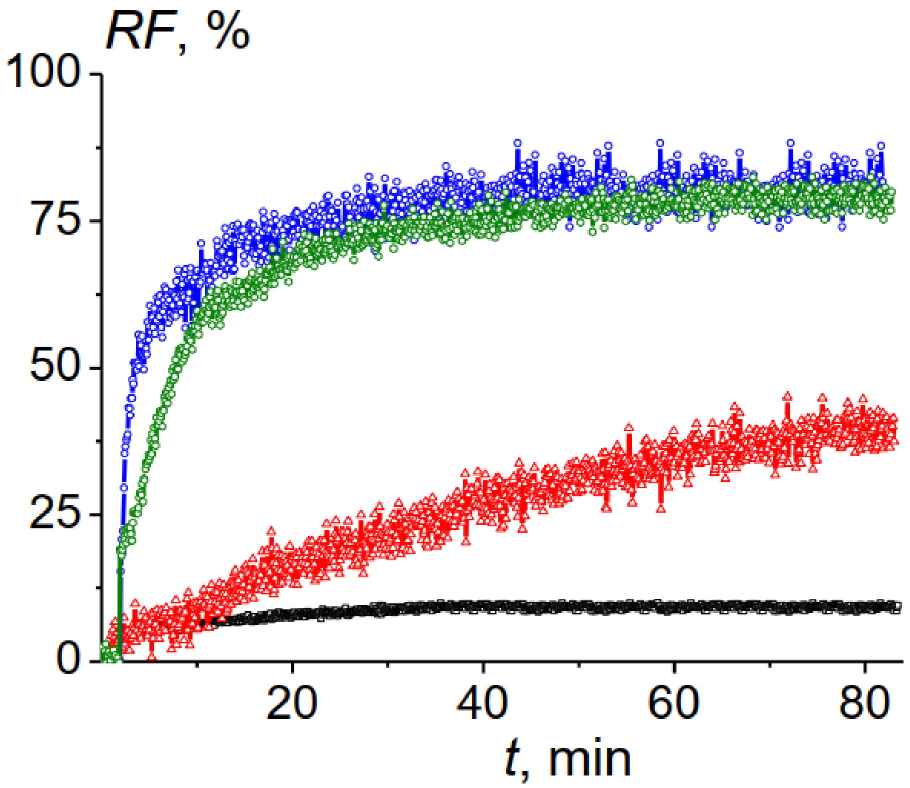

Figure S1. The dependence of relative fluorescence of calcein (***RF***) leaked from vesicles composed of DOPC (***black curve***), DOPC/DOPS (50/50 mol%) (***red curve***), DOPC/DOPG (50/50 mol%) (***green curve***) and DOPC/TOCL (50/50 mol%) (***blue curve***) on time (***t***). The lantibiotic was added into the liposomal suspension up to 0.1 mM at the zero time point.

Table S1. The parameters of exponential function fitting the time dependence of nisin-induced calcein
leakage from liposomes of different phospholipid composition.

lipid composition of vesicles	***RFmax, %***	***t0*, min***	***t1*, min****	***t2*, min****
DOPC	9 ± 3	10 ± 1		
DOPC/DOPS (50/50 mol%)	39 ± 3	58 ± 2		
DOPC/DOPG (50/50 mol%)	82 ± 2	-	0.8 ± 0.3	12 ± 2
DOPC/TOCL (50/50 mol%)	81 ± 2	-	4.8 ± 0.5	24 ± 2

According to the value of the χ_2-test, single(*)- or double(**)-exponential function was used to fit the time dependence of relative fluorescence of calcein leaked from vesicles with constants t_0 or t_1 and t_2 respectively.

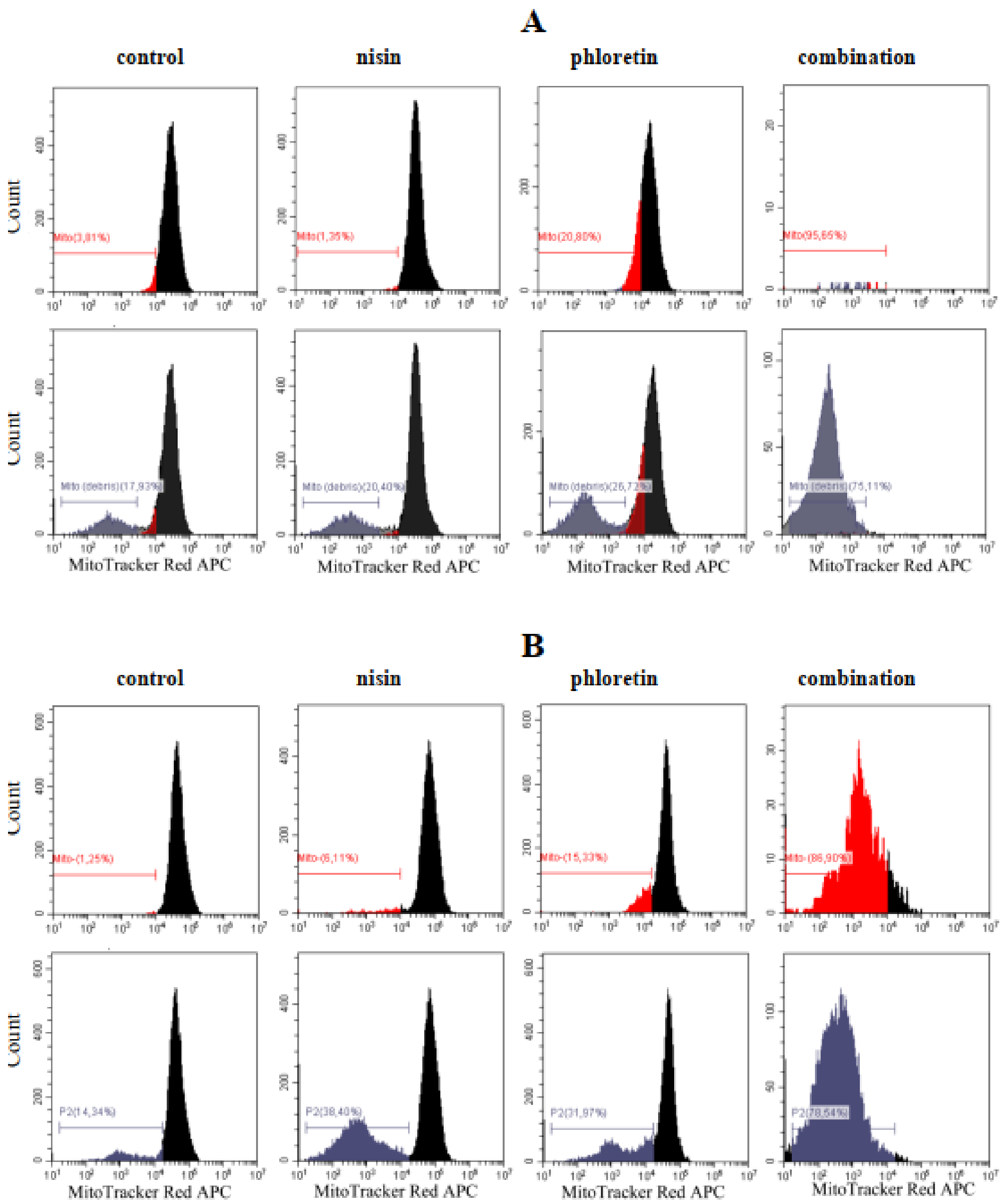

Figure S2. Histograms of the mitochondrial membrane potential of HepG2 cell line measured after 24 hours (**A**) and 48 hours (**B**) in the absence of any modifiers (control, ***firth column***) and in the incubation with 1 mM nisin (***second column***), 0.1 mM phloretin (***third column***) and their combination 1 mM nisin and 0.1 mM phloretin (***fourth column***). Vertical axis represents number of events, horizontal – fluorescent signal from the MitoTracker in APC (Allophycocyanin) channel. Red color signed "Mito" reflects the process of mitochondrial membrane depolarization, the black color is for cells with normal negative potential. Upper row of panel A and B represents cells excluding cell debris, lower row of panel A and B includes debris ("Mito(debris)"). Ten thousand events were recorded in total.
